# Conceptual model of adding antibiotics to dialysate fluid during renal replacement therapy

**DOI:** 10.1038/s41598-021-03450-1

**Published:** 2021-12-13

**Authors:** Ieva Bartuseviciene, Vaidas Vicka, Alvita Vickiene, Lidija Tetianec, Marius Dagys, Donata Ringaitiene, Andrius Klimasauskas, Jurate Sipylaite

**Affiliations:** 1 Faculty of Medicine, Vilnius University, Vilnius, Lithuania; 2 Clinic of Anaesthesiology and Intensive Care, Institute of Clinical Medicine, Faculty of Medicine, Vilnius University, Vilnius, Lithuania; 3 Clinic of Gastroenterology, Nephro-Urology and Surgery, Institute of Clinical Medicine, Faculty of Medicine, Vilnius University, Vilnius, Lithuania; 4 Department of Bioanalysis, Institute of Biochemistry, Life Sciences Center, Vilnius University, Vilnius, Lithuania

**Keywords:** Experimental models of disease, Renal replacement therapy

## Abstract

Studies have shown significant variability in antibiotic trough concentrations in critically ill patients receiving renal replacement therapy (RRT). The purpose of this study was to assess whether adding beta-lactam antibiotics to dialysate solution can maintain stable antibiotic concentrations during RRT in experimental conditions. A single compartment model reflecting the patient was constructed and connected to the RRT machine. Dialysate fluid was prepared in three different concentrations of meropenem (0 mg/L; 16 mg/L; 64 mg/L). For each dialysate concentration various combinations of dialysate and blood flow rates were tested by taking different samples. Meropenem concentration in all samples was calculated using spectrophotometry method. Constructed experimental model results suggest that decrease in blood meropenem concentration can be up to 35.6%. Moreover, experimental data showed that antibiotic loss during RRT can be minimized and stable plasma antibiotic concentration can be achieved with the use of a 16 mg/L Meropenem dialysate solution. Furthermore, increasing meropenem concentration up to 64 mg/L is associated with an increase antibiotic concentration up to 18.7–78.8%. Administration of antibiotics to dialysate solutions may be an effective method of ensuring a constant concentration of antibiotics in the blood of critically ill patients receiving RRT.

## Introduction

Acute kidney injury (AKI) is a common syndrome in patients admitted to the intensive care unit (ICU). The incidence of AKI in ICU patients varies widely from 3 to 33% with mortality ranging from 26 to 90%^1–8^. The cause of AKI is often multifactorial; however, sepsis, septic shock and concomitant systemic inflammatory response syndrome are the most common causes of AKI in the critically ill, accounting for over 60% of cases^9–13^. Furthermore, mortality in this subgroup of patients is considerably higher, reaching up to 50%^13^.

Irremissible treatment for these patients includes broad spectrum antibiotics and, in more severe cases, renal replacement therapy (RRT). A critical state and a diminished kidney function leads to altered distribution and elimination of the antibiotics. Upon starting the RRT these alterations are further exacerbated by introducing a machine generated clearance of drugs. Therefore, the antibiotic dosing during continuous RRT is particularly complex. Studies have shown that there is a significant variability in antibiotic trough concentrations in critically ill patients receiving RRT, revealing that empirical dosing failed to achieve the target in 25–60% of patients receiving continuous RRT^14–16^. This unintended under-dosing leads to increased resistance of the microflora and sub-optimal effect, prolonged hospitalization and worse clinical outcome^17–19^.

Beta-lactam antibiotics are used commonly in the management of critically ill patients with sepsis in ICU. Based on numerous in vitro and in vivo experimental data, the most significant parameter that influence their efficacy is the duration of the effective antibiotic exposure, i.e. time when antibiotic concentration in blood is higher than minimal inhibitory concentration (MIC) of the bacteria, in contrast to other possible pharmacokinetic targets^18,20–24^. Beta-lactam antibiotics are small and hydrophilic molecules, presenting with low to moderate distribution volumes, low protein binding and are mainly excreted by the kidneys^2,25^. Therefore, after initiation of the CRRT the clearance of these antibiotics is unpredictable, effected by constitution of the dialysate, blood and dialysate flow rates across the filtration membrane. The estimated clearance of beta-lactams during CRRT up to 30%^26^. Various dosing modifications are routinely applied to prevent inadequate antibiotic concentrations, leading to higher exposure to the drug, higher incidence of adverse effects and higher costs of the treatment^27^.

The aim of the study was to assess whether adding beta-lactam antibiotics to dialysate solution can maintain stable antibiotic concentrations during CRRT in experimental conditions.

## Materials and methods

### Construction of the experimental model

**Figure 1:**
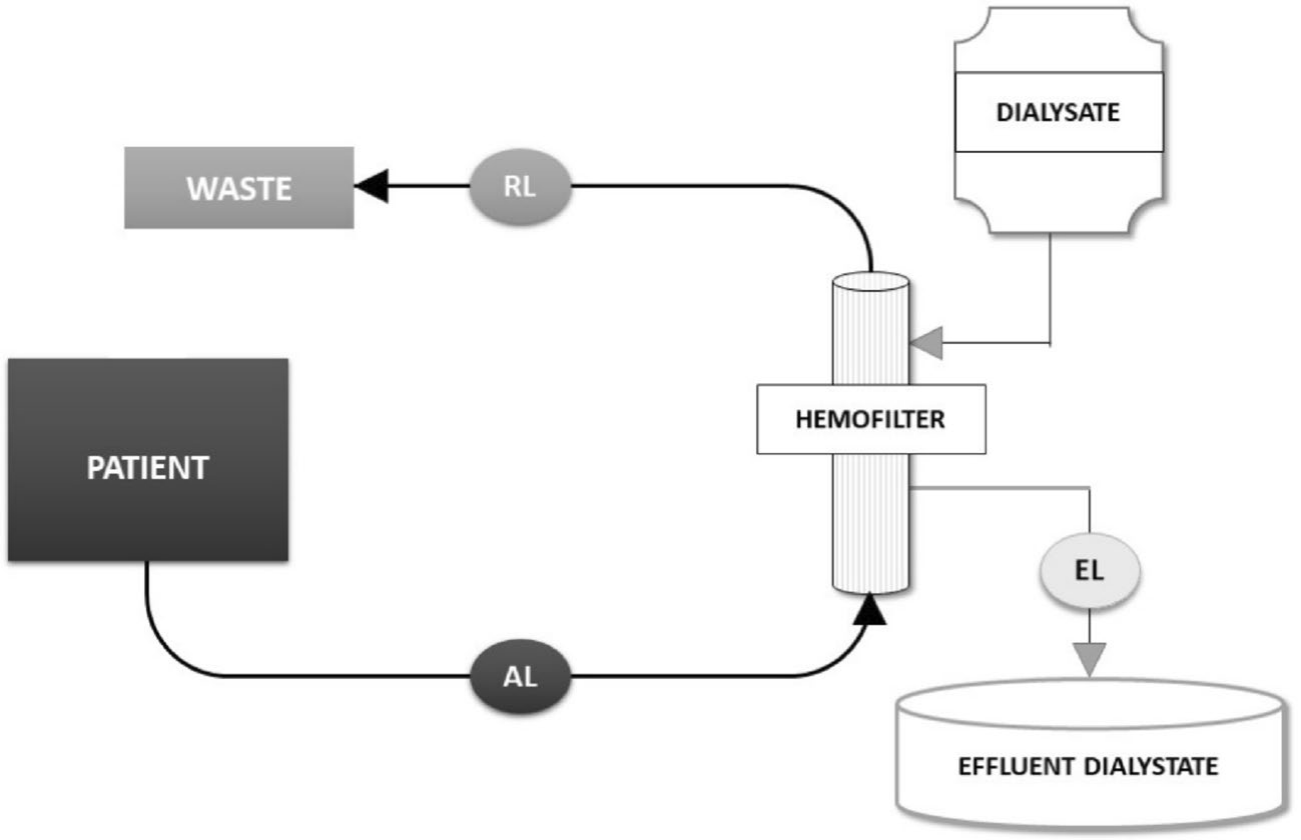
Schematics of the model. The schematics of the model, marking the sampling ports in the access line (AL), the return line (RL) and the effluent line (EL).

A single compartment model was constructed from the glass container of 25 L. The volume of the patient was imitated with a dialysate fluid with a fixed concentration of the drug (Table 1). This container was connected to the RRT machine. A modality of RRT chosen was continuous veno-venous hemodialysis (“MultifiltratePro”, Fresenius Medical Care Deutschland GmbH, Germany), representing the simplest process of drug movement in and out of patient's blood in the filter (“Kit Ci-Ca® HD 1000”, Fresenius Medical Care Deutschland GmbH, Germany). The same dialysate fluid was used for imitate the blood as well as for the dialysis itself, thus enabling the movement of only one molecule in the model—the drug. No real blood was used in the model, the term “blood” is used to refer to the dialysate in the circuit, in contrast to the dialysate in the dialysis compartment. Furthermore, no convection or ultrafiltration was used in the model. The effluent fluid was not recirculated back and directed straightly to the waste—creating and open model (Fig. 1).

**Table 1:** Composition of the dialysate.

Composite	Concentration
Sodium	133.0 mmol/L
Potassium	2.0 mmol/L
Magnesium	1.0 mmol/L
Chloride	116.2 mmol/L
Phosphate	0.8 mmol/L
Bicarbonate	20.0 mmol/L
Glucose	7.8 mmol/L
pH	7.8

Composition of the standard dialysate solution used in the study.

### Adding antibiotics to the container and the dialysate fluid

The beta-lactam antibiotic chosen for the study was meropenem (“Agenorem”, Medochemie LTD, Cyprus), due to its clinical indications and suitable pharmokinetic profile making it susceptible to elimination during CVVHD: volume of distribution of 21 L, 2% protein bound, molecular size of 383.5 Da, sieving coefficient of 0.93 and estimated removal of around 30% during the therapy^28^. However, the actual estimated sieving coefficient in our model was nearly 1.0 due to unobstructed movement of the drug and no clotting or clogging in the filter. The concentration of the meropenem set in the container was 16 mg/L, representing both recommended dosage for continuous infusion and the four-fold higher concentration than reported MIC of the most common microflora in the intensive care units^29^.

To determine whether adding meropenem into the dialysate fluid will remove the concentration gradient and equilibrate antibiotic concentrations during CVVHD the dialysate fluid was prepared in three different concentrations (0 mg/L; 16 mg/L; 64 mg/L). The maximum concentration we used was 64 mg/L, which is 2 times lower than concentration achieved after bolus injection of meropenem, therefore, in the safe range. Furthermore, the amount of meropenem used to achieve 64 mg/L in the dialysis bag was less than 1 g. Thus, we do comply with safety recommendations of European Medical Agency. The drug was injected into dialysate bags via a sterile accessory spike using a sterile plastic syringe. Each bag was then gently shaken to ensure proper mixing.

### Generating various blood and dialysate flow situations

To determine the effect of blood flow (Qb) and dialysate flow (Qd) three different sets of samples were generated, providing nine combinations of blood and dialysate flow rates. For each dialysate concentration (0 mg/L; 16 mg/L; 64 mg/L) various combinations of dialysate and blood flow were tested (Table 2). Samples from the returning line (RL) and effluent line (EL) were taken in every setting and stored in the refrigerator (4–8 °C) until laboratory test was made.

**Table 2:** Setting up the blood an dialysate flow.

	Q_B1_—100 ml/min	Q_B2_—150 ml/min	Q_B3_—200 ml/min
Q_D1_-1000 ml/h	Q_B1_Q_D1_	Q_B2_Q_D1_	Q_B3_Q_D1_
Q_D2_-2000 ml/h	Q_B1_Q_D2_	Q_B2_Q_D2_	Q_B3_Q_D2_
Q_D3_-3000 ml/h	Q_B1_Q_D3_	Q_B2_Q_D3_	Q_B3_Q_D3_

Nine different sets of different blood and dialysate flow rates were generated. Qb—blood flow (ml/min), Qd—dialysate flow (ml/min).

### Detection of meropenem

Meropenem concentration in all samples was calculated using spectrophotometry method at a wavelength of 300 nm using extinction coefficient. The natural degradation of the meropenem in the container was estimated by taking control samples on 0 time, 2 and 4 h later (C1, C2 and C3)^30^.

## Results

### Stability of the meropenem

**Figure 2:**
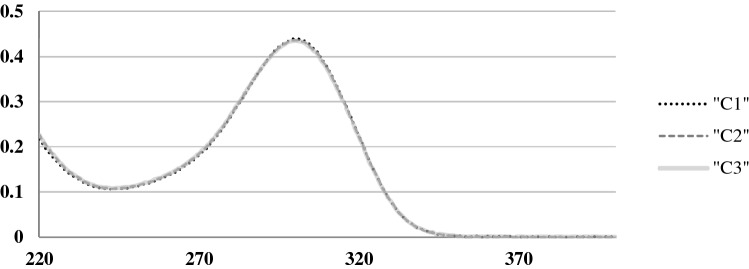
Absorption spectra of Meropenem. The figure demonstrates the absorption spectra of meropenem in controls (C1—time 0, C2—2 h, C3—4 h). No difference in absorption was registered. X axis—wavelength (nm), Y axis—absorbance.

All of Meropenem containing solutions were clear in appearance and no color change or precipitation was observed throughout the experiment period. Spectrophotometric testing of control samples from the container collected on 0 time, 2 and 4 h later (C1, C2 and C3) showed no significant changes in Meropenem concentration. The absorption spectra of Meropenem in controls are shown in Fig. 2.

### Effect of the meropenem added in the dialysate

**Figure 3:**
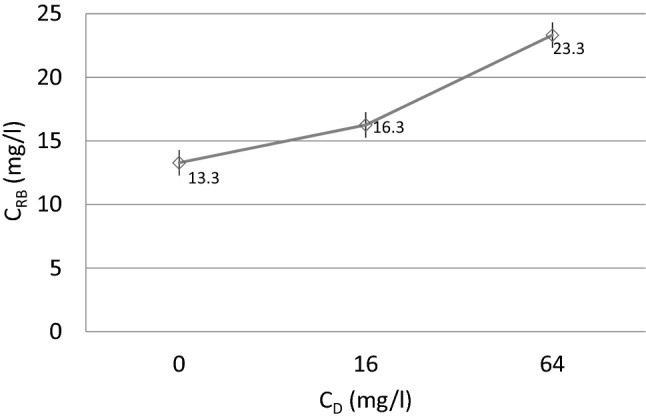
Meropenem concentration in returning blood flow. X-axis denotes the concertation of meropenem in dialysate solution; Y-axis denotes the concentration of meropenem in returning blood. The parameters during CVVHD were 150 ml/min of blood flow and 2000 ml/h of dialysate flow. CVVHD—continuous venovenous hemodialysis, C_RB_—concentration in returning blood, Cd—concentration in the dialysate. Error bars represent the confidence interval of 97% of a data set.

In the present model, a standard CVVHD procedure was associated with a decrease in blood meropenem concentration ranging from 8.8 to 35.6%, according to the flow rates of blood and dialysate. This loss was eliminated with the use of a 16 mg/L meropenem dialysate solution. Increasing meropenem concentration up to 64 mg/L in the dialysate solution revealed that this approach may raise antibiotic concentration up to 18.7–78.8% (Fig. 3).

### Effect of blood and dialysate flow rates

**Figure 4:**
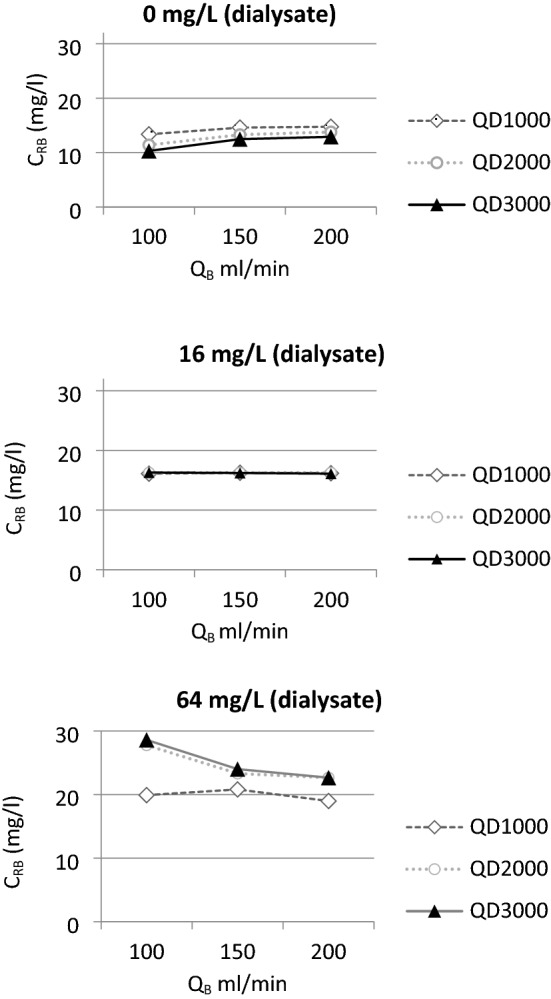
Meropenem concentration in returning blood—stratification according to the dialysate and blood flow rates. Three sets of different meropenem concentration in dialysate are presented. X-axis denotes the rate of the blood flow (ml/min), Y-axis denotes the concentration of meropenem in returning blood. QD denotes the rate of the dialysate flow (ml/hour); diamond, square and triangle denote the dialysate flow rates in in-graphic lines. C_RB_—concentration in returning blood.

The effects of blood and dialysate flow were less expressed on the returning blood concentration of meropenem than the concentration of meropenem in the dialysate fluid. Flow rate of dialysate was the main determinant of dialysis efficiency (Fig. 4). Increasing the rate of the dialysate flow leads to equilibration of the meropenem concentration in blood and dialysate (Fig. 4). In situations with no dialysate enrichment with meropenem this led to the decrease in meropenem concentration from 14.23% in 1000 ml/h flow to 11.89% in 3000 ml/h flow. In situations with dialysate enrichment with meropenem this lead to the increase in meropenem concentrations from 19.91% in 1000 ml/h flow to 25.1% in 3000 ml/h flow. To some extent this effect may be mitigated by increasing the blood flow and thus equilibrating towards the meropenem concentration in blood.

## Discussion

The results of this experimental study confirm the hypothesis that adding beta-lactam antibiotics to dialysate solution can maintain stable antibiotic concentrations during RRT. To our knowledge this is the first experimental study describing the antibiotic administration to dialysate in CRRT.

The study revealed that concentration of the antibiotic in the dialysate is the most important variable that determines the amount of antibiotic being lost during RRT. Our model suggests that the use of standard zero antibiotic concentration dialysate is associated with greater than 30% decrease in meropenem concentration during the procedure, leading to end concentration of 10 mg/L after dialysis, which has been shown to be not effective against the most common bacteria in ICU worldwide^29^. This has been already well demonstrated in previous trials, leading to recommendation to increase the dose of systemically administrated drug^31^. However, an increased intravenous antibiotic dose results in particularly enlarged risk of dose-related side effects as well as even higher concentration gradient of the drug between blood and dialysate. These pharmokinetic problems may be solved by eliminating the concentration gradient between blood and dialysate. In our study adding 16 mg/L of meropenem in dialysate eliminated the excretion of the drug. Based on this knowledge, the assumption can be made that stability of antibiotic concertation in plasma would be achieved more easily, when adding antibiotics in dialysate during CVVHD procedure.

The effects of flow settings on drug loss during RRT in our study are concordant to general principles of dialysis. The modality chosen in our study was CVVHD, proposing the most straightforward process of molecular diffusion across the filter membrane. Therefore, the effect of diffusion of meropenem in our model was mainly affected by the flow rate of the dialysate. Increasing the rate of the dialysate flow led to equilibration of the meropenem concentration in blood and dialysate, i.e., if the meropenem concentration in the dialysate was higher than in the incoming blood the outgoing blood would be more saturated with meropenem in higher dialysate flow conditions. To some extent this effect was mitigated by increasing the blood flow and thus equilibrating towards the meropenem concentration in blood. Since blood flow rates tested in our study were always much higher than the flow rates of the dialysate, there is no surprise, that the concentration of the effluent antibiotic was always more likely to resemble the concertation of antibiotic in the blood. Therefore, even when adding four times higher than necessary meropenem amount in dialysis fluid and decreasing the blood flow and increasing the dialysate flow to the maximum, an increase of meropenem concentration in returning blood was 30 mg/L. These results show, that it is both unpractical and not cost-efficient to try to increase the concentration of antibiotic in the returning blood via increasing the concentration of antibiotic in the dialysate.

Furthermore, the effect of the back filtration has not been fully addressed in the experimental model^32^. There are some limitations in the experimental model which favors the back filtration of the drug from the dialysate to the medium in the model, these are low flows in the circuit, no oncotic pressure or hematocrit in the circuit, low transmembrane pressure generated, and high flux filter used with no ultrafiltration. This is in part reflected in the experiment with low blood flow and a high dialysate flow providing high meropenem concentrations. These limitations have been in part addressed by trying to simplify the experimental model by using the same dialysate for the medium and for the dialysis, eliminating the effect of the oncotic pressure and hematocrit and by evaluating various setups of flow, thus generating different transmembrane pressures. These reflections must be considered if the results would be transferred to the clinical setting, which is not the purpose of this article. However, we can postulate that some amount of controlled back filtration, with somewhat diminished dialysis efficiency, increased risk of clotting may be allowed for the antibiotics to diffuse into the blood.

In the end, some other pharmokinetic variables were not considered in the experiment. Our model represented a situation where volume of distribution of meropenem is 25L. Based on pharmacokinetic data, Vd of meropenem is approximately 21L, indicating predominantly extracellular distribution. However, sepsis and other pathophysiological states result in fluid shifts from the intravascular compartment to the interstitial space. This leads to a decrease in the antibiotic plasma concentration by increasing the Vd of hydrophilic drugs. Furthermore, the degree of protein binding is a major determinant of the extent to which the drug is removed by RRT, predisposing that only an unbound drug can be removed. Based on pharmacokinetic data, meropenem protein binding should not exceed 2%, suggesting considerable net reduction of circulating drug during RRT. However, the degree of protein binding is influenced by many blood parameters. These blood (plasma) parameters vary widely during critical conditions. Studies have shown that the apparent plasma concentrations of bound and free Beta-lactam antibiotics are not in line with the expected values and a correlation with albumin concentration exists only in special conditions making it challenging to represent the system in a fully mechanistic fashion and thus underestimate the effect^18^.

Another limitation of the study is the permeability of the filter. During CRRT in vivo, blood is conducted through an extracorporeal circuit, activating coagulation by a complex interplay of patient blood and the circuit. Furthermore, critically ill patients may develop a procoagulant state due to multiple mechanisms including sepsis and SIRS related pro-inflammatory cytokine increase. High flux membranes with a cut-off of 30-40kD should be capable of eliminating significant amounts of inflammatory mediators including chemokines and cytokines by convection or by direct absorption on the filter. This advantage of RRT technique is effectively used in the management of sepsis related cytokinemia^33^. However, an increase in cytokine clearance result in decreased filter membrane permeability. Our model represented a ~ 0% reduction in permeability during CVVHD procedure. Changes in membrane permeability may lead to significant inadequacy between experimental design and clinical application.
